# Assessment of financial screening and navigation capabilities at National Cancer Institute community oncology clinics

**DOI:** 10.1093/jncics/pkad055

**Published:** 2023-08-10

**Authors:** Ari Bell-Brown, Kate Watabayashi, Debbie Delaney, Ruth C Carlos, Shelby L Langer, Joseph M Unger, Riha R Vaidya, Amy K Darke, Dawn L Hershman, Scott D Ramsey, Veena Shankaran

**Affiliations:** Hutchinson Institute for Cancer Outcomes Research, Fred Hutchinson Cancer Center, Seattle, WA, United States; Hutchinson Institute for Cancer Outcomes Research, Fred Hutchinson Cancer Center, Seattle, WA, United States; Hutchinson Institute for Cancer Outcomes Research, Fred Hutchinson Cancer Center, Seattle, WA, United States; Department of Radiology, University of Michigan Medical Center, Ann Arbor, MI, United States; Center for Health Promotion and Disease Prevention, Edson College of Nursing and Health Innovation, AZ State University, Phoenix, AZ, United States; Division of Public Health Sciences, Fred Hutchinson Cancer Center, Seattle, WA, United States; SWOG Statistics and Data Management Center, Seattle, WA, United States; Division of Public Health Sciences, Fred Hutchinson Cancer Center, Seattle, WA, United States; SWOG Statistics and Data Management Center, Seattle, WA, United States; SWOG Statistics and Data Management Center, Seattle, WA, United States; Division of Hematology/Oncology, Columbia University, New York, NY, United States; Hutchinson Institute for Cancer Outcomes Research, Fred Hutchinson Cancer Center, Seattle, WA, United States; Hutchinson Institute for Cancer Outcomes Research, Fred Hutchinson Cancer Center, Seattle, WA, United States; Division of Hematology, University of Washington, Seattle, WA, USA, United States

## Abstract

**Background:**

Cancer-related financial hardship is a side effect of cancer diagnosis and treatment, and affects both patients and caregivers. Although many oncology clinics have increased financial navigation services, few have resources to proactively provide financial counseling and assistance to families affected by cancer before financial hardship occurs. As part of an ongoing randomized study testing a proactive financial navigation intervention, S1912CD, among sites of the National Cancer Institute Community Oncology Research Program (NCORP), we conducted a baseline survey to learn more about existing financial resources available to patients and caregivers.

**Methods:**

The NCORP sites participating in the S1912CD study completed a required 10-question survey about their available financial resources and an optional 5-question survey that focused on financial screening and navigation workflow and challenges prior to starting recruitment. The proportion of NCORP sites offering financial navigation services was calculated and responses to the optional survey were reviewed to determine current screening and navigation practices and identify any challenges.

**Results:**

Most sites (96%) reported offering financial navigation for cancer patients. Sites primarily identified patients needing financial assistance through social work evaluations (78%) or distress screening tools (76%). Sites revealed challenges in addressing financial needs at the outset and through diagnosis, including lack of proactive screening and referral to financial navigation services as well as staffing challenges.

**Conclusions:**

Although most participating NCORP sites offer some form of financial assistance, the survey data enabled identification of gaps and challenges in providing services. Utilizing community partners to deliver comprehensive financial navigation guidance to cancer patients and caregivers may help meet needs while reducing site burden.

##  

Cancer-related financial hardship, often referred to as “financial toxicity,” is a well-documented side effect of cancer diagnosis and treatment, with a growing body of literature suggesting that financial difficulty affects at least 30%-50% of all cancer patients (1-3). Compared with individuals without cancer, cancer patients face higher rates of out-of-pocket health care spending, medical debt, and bankruptcy (1,3-10). The experience of financial hardship is often shared with spouse and partner caregivers who are not only directly affected by increased household medical spending, but also may need to take time off from work to perform caregiver duties (11-16). In recent years, many oncology clinics have improved their capacity to provide financial counseling and support for families who are struggling financially. However, very few oncology clinics have enough resources to *proactively* assist families with medical costs and counsel households with regard to management of assets, debts, and expenses before their financial status deteriorates (17-19). Given the financial impact of a cancer diagnosis on both patients and caregivers, there is a critical need to develop and test proactive interventions aimed at reducing household financial hardship.

The CREDIT study (A Randomized Trial Addressing Cancer-Related Financial Hardship Through Delivery of a Proactive Financial Navigation Intervention; S1912CD) is a prospective randomized controlled trial that evaluates whether a proactive financial navigation program helps cancer patients and their spouse or partner caregivers understand and manage the financial aspects of cancer care to avoid financial hardship and downstream impacts on health and well-being. This study is being conducted through the NCI Community Oncology Research Program (NCORP) network, which brings cancer care delivery research (CCDR) to over 900 community oncology practices throughout the country (20). A 2017 landscape survey of financial navigation practices at NCORP clinics found that 72% of participating sites reported a financial screening process, but only 50% of sites had a cancer-specific financial navigator (21). This landscape survey was administered again in 2022, with similar results (22).

Although the landscape survey provides general insights into financial screening and navigation practices at NCORP sites, it includes only 3 general questions related to financial navigation. Thus, to gain a more in-depth understanding of financial resources available across NCORP clinics, the CREDIT team administered an in-depth survey (“site survey”) to NCORP sites participating in CREDIT prior to the start of site recruitment to the intervention. In this article we summarize the findings from the baseline CREDIT site survey and describe existing financial resources available to cancer patients and their caregivers at NCORP sites. These findings are intended to help researchers, clinicians, and oncology practices understand gaps in available financial resources and identify opportunities for quality improvement and research interventions that can comprehensively address cancer-related financial hardship.

## Methods

### Setting

The CREDIT (S1912CD) study is led by the SWOG Cancer Research Network and conducted through the NCORP, a national network of research bases and community sites bringing cancer clinical trials and care delivery studies to community settings (20). Community sites consist of consortiums of researchers, public hospitals, physician practices, academic medical centers, and other groups providing health care services in communities (20). Fourteen NCORP-designated community sites are categorized as minority and underserved (MU-NCORP) sites, serving patient populations comprising at least 30% racial/ethnic minorities or rural residents. The CREDIT study was activated on July 26, 2021, and is actively enrolling patients at the time of this report.

### Site survey design and analysis

A baseline site survey was administered to all sites prior to the start of recruitment. The protocol for S1912CD includes a site eligibility stipulation stating that sites must complete the survey in order to register patients to the study in the Oncology Patient Enrollment Network (OPEN). Any staff member at a participating site is able to fill out the baseline survey. Sites were asked to access and complete the site survey via the REDCap (Research Electronic Data Capture) system and upload a certificate of survey completion to the CTSU (Cancer Trials Support Unit) regulatory submission portal to be eligible to register study participants. The study was reviewed and approved by the National Cancer Institute Central Institutional Review Board. The baseline site survey (Supplementary Methods: Survey 1, available online) consists of 10 questions relating to available financial navigation resources for cancer patients at the institution and includes questions such as whether the site offers financial assistance, how patients are screened for financial hardship, what types of financial services are offered, and primary reasons that financial assistance is requested. NCORP affiliate sites that shared the same financial resources were able to fill out 1 survey for all sites as long as they listed each site name and their unique cancer therapy evaluation program identification (CTEP ID) in the survey. After the baseline survey, sites were given an optional additional 5-question survey (Supplementary Methods: Survey 2, available online) focused specifically on their financial screening and navigation challenges and workflow, and sites were also allowed to provide open-ended responses. Survey responses submitted by August 31, 2022, were analyzed for this article.

We evaluated the surveys by individual site, using the CTEP ID to identify each unique site. Here, we report the proportions of sites offering financial navigation, types of financial assistance offered, how patients are identified and referred for financial navigation services, and the primary reasons cancer patients request financial assistance. The baseline survey provided the following definition for financial navigator:

A person or team who works with patients and their families to help them reduce stress or hardship related to the cost of treatment for a medical condition, such as cancer. Financial navigation helps patients understand their out-of-pocket expenses and what their health insurance plans may cover. Financial navigation may also help patients set up payment plans, find cost-saving methods for treatments, and improve access to health care services that the patient needs. Financial navigation services may be provided by a dedicated person (a financial navigator), or by positions such as social workers, billing staff, or practice providers.

Sites were able to choose multiple options for questions 1, 4, 5, and 10 in the baseline site survey. Results are reported for all participating NCORPs. We calculated *z*-scores to determine if statistically significant differences were observed in services offered between MU-NCORPs and non–MU-NCORPs in recognition that a larger portion of the patient population at MU-NCORPs is medically underserved and as a result those sites may offer unique or more extensive services to patients experiencing financial hardship compared to the non–MU-NCORPs patient population.

## Results

**Figure 1.**
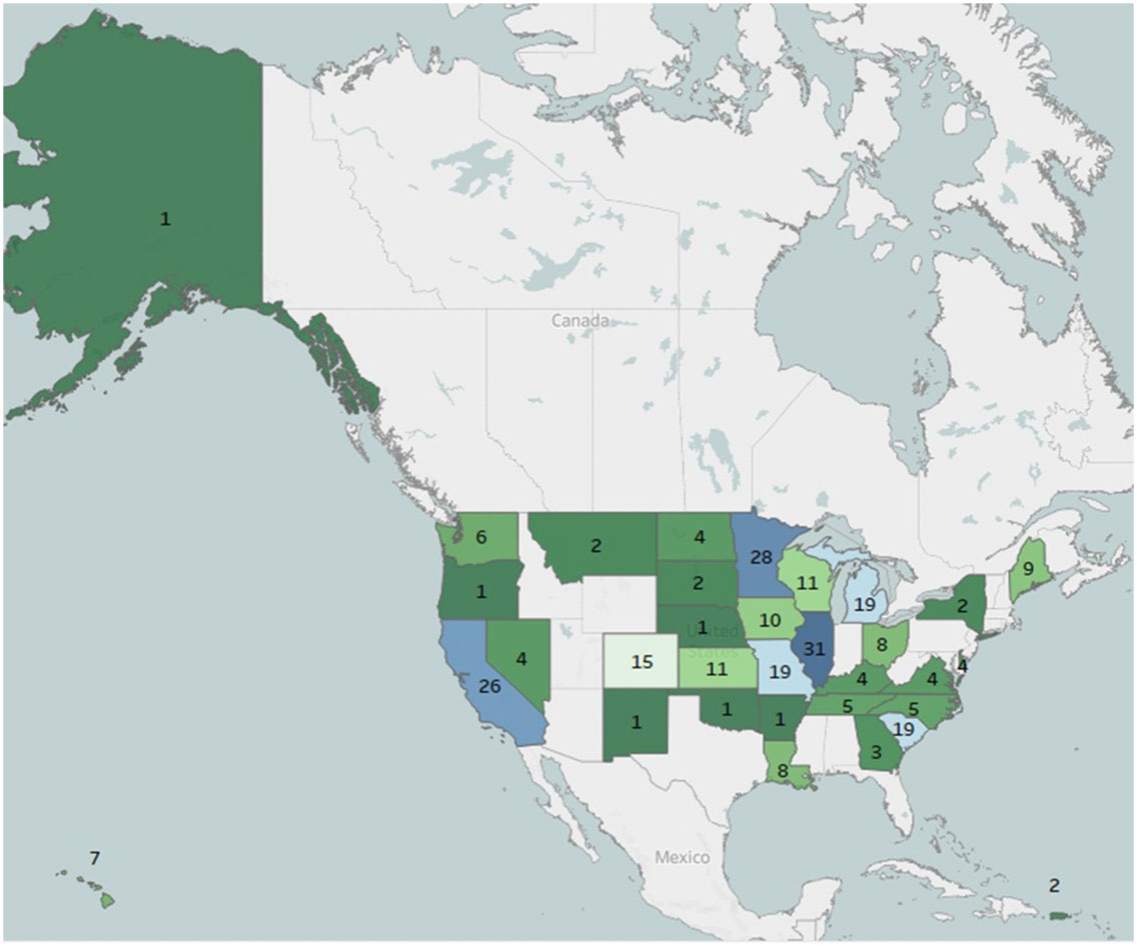
Participating sites by state.

Between May 2021 and August 2022, 274 individual NCORP practices completed the survey, of which 38 were MU-NCORPs. Figure 1 shows the number and geographic distribution of participating sites. Sites were distributed across the United States, with the largest clusters of sites in Illinois (n = 31), Minnesota (n = 28), and California (n = 26). The majority of participating sites provided comprehensive cancer care (n = 221, 81%), 35 sites (13%) provided medical oncology, and the remaining sites provided either radiation, surgical, or a combination of treatment services. Most participating sites are community medical centers (n = 236, 86%), with 38 (14%) academic medical centers. A majority of participating MU-NCORP sites (n = 23, 61%) are academic medical centers, whereas only 15 (6%) of participating non–MU-NCORP sites are academic medical centers. Staff members completing the survey included clinical research associates or coordinators (n = 75, 27%), research or program managers (n = 51, 19%), research nurses (n = 48, 18%), regulatory specialists (n = 36, 13%), role unknown (n = 26, 9%), research directors (n = 20, 7%), research administrators (n = 15, 5%), and principal investigators (n = 3, 1%).

Most sites (n = 262, 96%) reported that they offer some type of financial navigation for cancer patients at their site, with 87% of MU-NCORP sites offering financial navigation and 97% of non–MU-NCORP sites offering financial navigation (*P* = .01). Table 1 shows results from the site survey, including how cancer patients who need financial assistance are identified, how financial navigation is delivered, and what types of financial assistance are offered. The majority of sites reported using social work evaluations (n = 214, 78%) or distress-screening tools (n = 209, 76%) to identify patients in need of financial assistance. Non MU-NCORP sites were more likely than MU-NCORP sites to use distress screening tools (79% vs 61% respectively, *P* = .02). MU-NCORP sites were more likely than non–MU-NCORP sites to identify cancer patients needing financial assistance at multiple time points (84% vs 64%, *P* = .02). Most sites (n = 176, 64%) reported offering financial navigation to all cancer patients, and MU-NCORP sites were more likely to offer financial navigation to underinsured cancer patients (8% vs 2%, *P* = .04). Sites primarily provide financial navigation using a financial counselor/navigator (n = 204, 74%) and non–MU-NCORP sites were more likely to use social work to provide financial navigation (55% vs 34%, *P* = .02). Sites reported assisting patients with both medical related (n = 266, 97%) and non–medical related (n = 264, 96%) financial issues that arise due to cancer diagnosis. Primary reasons that cancer patients request financial assistance were high across all categories (pharmaceutical assistance, help paying for care, help understanding bills, and help paying for nonmedical costs), and thus comprehensive financial assistance for cancer patients may be necessary to avoid financial hardship.

**Table 1.** Site survey results

Survey question	MU-NCORP sites	Non–MU-NCORP sites	*P*	All sites
	(n = 38)	(n = 236)		(n = 274)
Do you offer financial navigation services?				
Yes	33 (87%)	229 (97%)	.01^b^	262 (96%)
No	5 (13%)	7 (3%)	.01^b^	12 (4%)
How are cancer patients who need financial assistance identified?^a^				
Social work evaluation	28 (71%)	186 (79%)	.49	214 (78%)
Distress screening tool(s)	23 (61%)	186 (79%)	.02^b^	209 (76%)
Patient intake form(s)	16 (42%)	94 (40%)	.82	110 (40%)
Other	11 (29%)	98 (42%)	.13	109 (40%)
Electronic health record	12 (32%)	86 (36%)	.63	98 (36%)
Clinic team form(s)	3 (8%)	28 (12%)	.47	31 (11%)
No method	1 (3%)	9 (4%)	.76	10 (4%)
When are cancer patients who need financial assistance typically identified?				
Multiple time points	32 (84%)	152 (64%)	.02^b^	184 (67%)
First clinic visit	5 (13%)	67 (38%)	.00^b^	72 (26%)
No answer	1 (3%)	9 (4%)	.76	10 (4%)
During treatment	0 (0%)	8 (3%)	.28	8 (3%)
Financial navigation is offered to:				
All cancer patients	23 (61%)	153 (65%)	.63	176 (64%)
Only patients who request it	3 (8%)	46 (19%)	.10	49 (18%)
Other	4 (11%)	25 (11%)	1	29 (11%)
Financial navigation is not offered	5 (13%)	7 (3%)	.01^b^	12 (4%)
Underinsured cancer patients	3 (8%)	5 (2%)	.04^b^	8 (3%)
Financial navigation at our clinic is primarily provided by:^a^				
Financial counselor or navigator	24 (63%)	180 (76%)	.09	204 (74%)
Social work	13 (34%)	130 (55%)	.02^b^	143 (52%)
Billing staff	5 (13%)	59 (25%)	.11	64 (23%)
Nurse navigator	12 (32%)	36 (15%)	.01^b^	48 (18%)
Clinic website (resource list)	3 (8%)	39 (17%)	.16	42 (15%)
Other	3 (8%)	35 (15%)	.25	38 (14%)
Financial navigation is not offered	5 (13%)	7 (3%)	.01^b^	12 (4%)
Practice provider	2 (5%)	6 (3%)	.52	8 (3%)
Our clinic assists patients with medical related (ie, insurance, copays, drug costs) financial issues that arise due to cancer diagnosis:				
Yes	37 (97%)	228 (97%)	1	266 (97%)
No	1 (3%)	7 (3%)	1	8 (3%)
Our clinic assists patients with non–medical related (ie, housing, income loss, transportation) financial issues that arise due to cancer diagnosis:				
Yes	36 (95%)	228 (97%)	.52	264 (96%)
No	2 (5%)	8 (3%)	.52	10 (4%)
What is the primary reason(s) cancer patients request financial assistance?^a^				
Pharmaceutical assistance	30 (79%)	197 (83%)	.55	227 (83%)
Help understanding bills and/or out-of- pocket costs	34 (89%)	188 (80%)	.19	222 (81%)
Help paying for care	32 (84%)	178 (75%)	.23	210 (77%)
Help paying for nonmedical costs	31 (82%)	173 (73%)	.24	204 (76%)
Other	8 (21%)	29 (12%)	.13	37 (14%)

a Respondents can select more than one answer. NCORP = National Cancer Institute Community Oncology Research Program; MU = minority/underserved.

b *P* <. 05, which indicates statistical significance.

**Figure 2.**
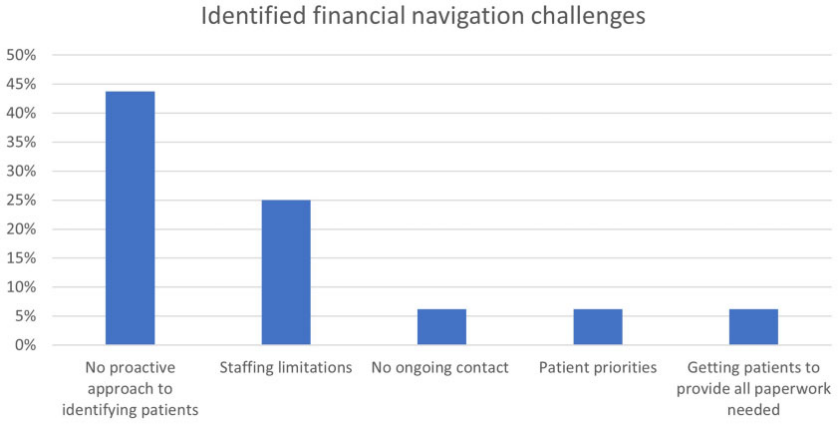
Challenges to providing financial navigation identified by sites (n = 16).

Although the overwhelming majority of sites offer some type of financial navigation services for their patients, sites completing the supplemental survey (n = 16) revealed challenges in the identification and referral of patients as well challenges in staffing the high volume of referrals (Figure 2). Nearly half of sites reported that they lacked a system for proactively identifying the financial needs and concerns of the patients they serve.

## Discussion

Although most of the NCORP sites participating in the CREDIT study offer *some* form of financial assistance or navigation, many gaps and challenges were identified. First, many sites have limited staffing and are unable to provide proactive assistance to all clinic patients, relying instead on providers’ referrals or patients’ direct requests for assistance. Our results are similar to those reported in prior studies showing that very few oncology clinics, and cancer centers are able to provide assistance with medical costs and counsel families about management of assets, debts, and household expenses before they experience financial toxicity (17-19,21). The following response from one site may, indeed, reflect a shared experience across NCORPs: “Unfortunately, these services are time intensive and require a great deal of training and expertise to appropriately address each patient’s individual concerns. As of present the cancer center has employed only 3 social workers and 1 financial advocate to address a large regional cancer center… This results in a chasm of unmet needs and potentially unaddressed barriers.”

Another barrier clinics face in providing timely financial assistance is systematically identifying patients who could benefit from these services. Despite the majority of sites reporting that they have a process in place to screen patients for financial hardship, nearly half (44%) of those sites responding to the supplemental survey said that proactive approaches to identifying and screening patients was a challenge. This finding suggests that simply having a screening process or tool available at a clinic is not enough to guarantee that patients can successfully access financial support services. There could be several reasons for this potential gap. The majority of sites surveyed rely on either social work referrals or distress screening tools to identify patients with financial needs. Social work referrals are limited by staffing, the second most reported challenge. The effectiveness of screening tools is limited by factors such as the timing and method of administration, eg, are patients being screened at the optimal time, is screening conducted in a private space, does it require patients to have access to an online form or portal, and are questions available in languages other than English? Sites that reported the use of the distress-screening tool and also reported challenges in proactive approaches to identifying patients facing financial hardship also reported issues such as screening not being done consistently, patients not being transparent around needs, and time and/or staffing difficulties in reviewing and responding to the distress screening tool. These issues are critical, particularly difficulties in identifying patients, which warrants further study because if patients cannot be identified, they cannot access the financial assistance services that sites reported they can offer downstream in their clinic workflow.

We acknowledge the limitations of this study. Although the surveys represent participating sites in the CREDIT study, the results are not reflective of all NCORP sites, and it is possible that other sites have different approaches to financial hardship screening and services provided to counteract financial hardship for oncology patients. Next, this study does not explore the extent to which reported financial screening and navigation services at the sites actually translate into meaningful and timely assistance for the patients and families they serve or whether patients and families utilize available services when offered. In other words, presence of a financial navigator does not guarantee successful financial navigation. Additionally, response to our optional follow-up survey that provided more in-depth information around site workflows for identifying and responding to financial hardship was very small.

Despite the noted limitations, we feel that the results from this survey support the need to identify and test more robust approaches to addressing financial hardship among cancer patients. Given the evidence showing that financial hardship can negatively affect quality of life and survival, as well as result in more intense hospital-based care (1-3,23), finding comprehensive approaches to addressing financial hardship for patients and caregivers affected by a cancer diagnosis is critically important. Results from this survey highlight some gaps in the ability of NCORP sites to address financial concerns at the outset and through cancer diagnosis and treatment. The CREDIT study (S1912CD) will evaluate our hypothesis that a successful financial navigation intervention implemented at the household level could result in improvements in financial, psychosocial, and clinical outcomes for patients and their caregivers.

A landscape survey of financial navigation services offered by NCORP sites showed that while sites by and large offer some form of financial navigation, gaps in services at key points such as screening and referral to navigation exist. In some cases these could be filled by outside organizations, reducing the staffing and time burden on clinical sites themselves. Findings from these surveys provide support for the potential utility of comprehensive proactive financial navigation and follow-up systems to mitigate financial toxicity for patients coping with cancer and their caregiving partners.

## Supplementary Material

pkad055_Supplementary_Data

## Data Availability

Deidentified datasets will be made available following appropriate terms and conditions upon request to the corresponding author.
