# Supplementary material for: Assessment of financial screening and navigation capabilities at National Cancer Institute community oncology clinics
Source: JNCI Cancer Spectr. 2023 Aug 10;7(5):pkad055. doi: 10.1093/jncics/pkad055 (PMC10471524; doi:10.1093/jncics/pkad055)
Supplement: pkad055_Supplementary_Data [file pkad055_supplementary_data.pdf]

SUPPLEMENTARY MATERIAL

Supplementary Methods: Survey One

S1912CD: ADDRESSING CANCER-RELATED FINANCIAL HARDSHIP THROUGH DELIVERY OF A PROACTIVE FINANCIAL NAVIGATION INTERVENTION

To: NCI Community Oncology Research Program (NCORP) Site Staff

Survey Purpose: To understand what (if any) financial navigation resources are available for cancer patients at your institution.

Please answer these questions as completely as possible.

Instructions:

A. If your sites share the same financial navigation resources, you may fill out 1 survey for multiple sites. List each site name and each CTEP Site Code on the first page of the survey separated by a comma.

   Example:

   NCORP affiliate(s) or sub-affiliate name(s): Site Name 1, Site Name 2, Site Name 3, etc.

   NCORP affiliate(s) or sub-affiliate CTEP Site Code(s): CTEP Site Code 1, CTEP Site Code 2, CTEP Site Code 3, etc.

B. Affiliated sites that do not share financial navigation resources must *each* complete a survey.

NCORP affiliate(s) or sub-affiliate name(s):
NCORP affiliate(s) or sub-affiliate CTEP Site Codes(s):

Email address where the Site Survey Completion Certificate is to be sent:

Financial navigator defined:

A person or team who works with patients and their families to help them reduce stress or hardship related to the cost of treatment for a medical condition, such as cancer. Financial navigation helps patients understand their out-of-pocket expenses and what their health insurance plans may cover. Financial navigation may also help patients set up payment plans, find cost-saving methods for treatments, and improve access to healthcare services that the patient needs. Financial navigation services may be provided by a dedicated person (a financial navigator), or by positions such as social workers, billing staff, or practice providers.

S1912CD Site Implementation Survey

1. How are cancer patients who need financial assistance identified? (Select all that apply)
   - o Social Work Evaluations
   - o Distress Screening Tools
   - o Clinic Team Forms
   - o Patient Intake Forms
   - o Electronic Health Records
   - o Other methods of identification
   - o Our site has no method to identify patients needing financial assistance (skip to Q3)

2. When are cancer patients who need financial assistance typically identified at your clinic?
   - o At the first clinic visit
   - o During treatment
   - o After treatment
   - o At multiple time points
   - o Our site has no method to identify patients needing financial assistance

3. Financial navigation is offered to:
   - o All cancer patients regardless of insurance/financial status
   - o Cancer patients that are underinsured (Medicaid or no insurance)
   - o Only patients that request financial assistance
   - o Other (Please specify): _____________________
   - o Financial navigation is not offered at our site

4. Financial navigation at our clinic is primarily provided by: (Select primary method(s))
   - o A social worker
   - o A financial counselor
   - o Clinic website list of resources
   - o Billing staff
   - o Practice providers
   - o Nurse navigator
   - o Financial navigator
   - o Other (Please specify): ___________________
   - o Financial navigation is not provided at our site

5. Our clinic offers financial navigation services in the following languages: (Select all that apply)
   - o English
   - o Spanish
   - o Other (Please specify): ___________________
   - o Financial navigation services are not offered at our site

6. Our clinic assists patients with medical related (i.e. insurance, co-pays, drug costs) financial issues that arise due to cancer diagnosis.
   - o No
   - o Yes (Please list types available in comment box below.)

   Comment box: __________________________

7. Our clinic assists patients with non-medical related (i.e. housing, income loss, transportation) financial issues that arise due to cancer diagnosis.
   - o No
   - o Yes (Please list types available in comment box below.)

   Comment box: __________________________

8. Our clinic advertises financial services through written materials in the waiting and exam rooms.
   - o No
   - o Yes

9. If a cancer patient indicates that they need financial assistance, the standard response provided by our clinic is:
   - o Refer patient to social work
   - o Refer patient to in house financial counselor or financial assistance services
   - o Refer patient to outside financial assistance program (Please specify which external partners you work with) ____
   - o There is no standard at our clinic if a patient indicates they need financial assistance
   - o Other (Please specify): ___________________________

10. What is the primary reason(s) cancer patients request financial assistance at your clinic? (Select all that apply)
   - o Need pharmaceutical assistance
   - o Need help paying for care
   - o Need help understanding medical bills and out of pocket costs
   - o Need help paying for non-medical care costs
   - o Other (Please specify): ____________________________

Thank you for completing this study.

Supplementary Methods: Survey Two

S1912CD Site Implementation Survey- Supplemental Questions

1. Please provide more details around your process to identify cancer patients to refer for financial assistance/navigation?
2. Are there certain criteria you use to identify patients in need of financial assistance or navigation?
3. Can you tell me about how patients are referred for financial navigation?
4. How does your clinic identify what types of financial assistance a patient needs? How is financial assistance delivered?
5. Are there any challenges with your current process for identifying and referring patients for financial navigation? Are there parts of your current process you feel are working well?
